# When Tuberculosis Bleeds: Intestinal Tuberculosis Presenting With Hematochezia

**DOI:** 10.7759/cureus.106399

**Published:** 2026-04-03

**Authors:** James M Day

**Affiliations:** 1 Internal Medicine, U.S. Naval Hospital Guam, Agana Heights, GUM

**Keywords:** extra-pulmonary tuberculosis (eptb), gastrointestinal tuberculosis, guam, hematochezia, intestinal tuberculosis

## Abstract

Intestinal tuberculosis is an uncommon but clinically significant manifestation of tuberculosis that often presents with nonspecific gastrointestinal symptoms. Although the ileocecal region is most frequently involved, overt gastrointestinal bleeding is an unusual presentation, particularly in elderly patients who may lack classic constitutional symptoms. An elderly man presented to a community hospital in Guam with hematochezia one week after the diagnosis of pulmonary tuberculosis and initiation of rifampin, isoniazid, pyrazinamide, and ethambutol. Colonoscopy revealed a large semi-circumferential ulcer in the terminal ileum with a non-bleeding visible vessel. Histopathologic evaluation demonstrated necrotizing granulomatous inflammation, and acid-fast bacilli staining confirmed mycobacterial infection, establishing the diagnosis of intestinal tuberculosis. Despite appropriate anti-tuberculous therapy, the presence of concurrent pulmonary and intestinal disease reflected an advanced disease burden and poor prognosis. Given advanced frailty and clinical status, the patient and his family elected to pursue comfort-focused care. This case highlights intestinal tuberculosis as an uncommon cause of gastrointestinal bleeding and emphasizes its diagnostic overlap with conditions such as Crohn’s disease. It underscores the importance of biopsy in granulomatous ileal disease and the need to maintain clinical suspicion for tuberculosis, particularly in patients from endemic regions or with relevant epidemiologic risk factors.

## Introduction

Intestinal tuberculosis is an uncommon but clinically significant manifestation of tuberculosis that poses substantial diagnostic challenges because of its nonspecific presentation and overlap with more common gastrointestinal conditions. Extrapulmonary tuberculosis accounts for approximately 15%-20% of cases in immunocompetent individuals; however, an additional 23%-30% of cases involve concurrent pulmonary and extrapulmonary disease, and such cases are classified separately rather than as extrapulmonary-only disease in surveillance systems. Intestinal involvement represents only a small fraction of cases, even in endemic regions such as the Philippines, where tuberculosis incidence is estimated at approximately 554-600 cases per 100,000 population [1,2]. The ileocecal region is most frequently affected, likely due to prolonged fecal stasis, abundant lymphoid tissue, and increased absorptive capacity [3,4].

Classic symptoms reported in observational cohorts include chronic abdominal pain, weight loss, fever, and altered bowel habits, whereas gastrointestinal hemorrhage is a relatively uncommon complication, occurring in approximately 3%-14% of complicated cases, making overt bleeding presentations unusual [5]. Crohn’s disease represents the primary non-infectious diagnostic mimic of intestinal tuberculosis, as both can present with granulomatous inflammation, transmural involvement, and overlapping clinical features. Older adults with tuberculosis may present atypically, often lacking constitutional symptoms, which reduces clinical suspicion and contributes to diagnostic delay [6-9]. Clinically, this case demonstrates an uncommon presentation of intestinal tuberculosis in an elderly patient with concurrent pulmonary involvement and hematochezia. Diagnostically, it emphasizes the importance of maintaining a broad differential diagnosis informed by regional epidemiology when ileal granulomas are identified.

## Case presentation

An elderly man in his late 80s, born in the Philippines and residing in Guam for more than 50 years, presented to a federally funded community hospital with hematochezia. One week prior, he had been hospitalized and newly diagnosed with active pulmonary tuberculosis, for which he was started on rifampin and isoniazid, along with renally dosed pyrazinamide and ethambutol. His past medical history was notable for chronic obstructive pulmonary disease managed with inhalers without home oxygen and decades of tobacco use. He was not taking anticoagulants, antiplatelet agents, or nonsteroidal anti-inflammatory drugs. His bacillus Calmette-Guérin (BCG) vaccination status was unknown. On review of systems, he reported generalized weakness but denied weight loss, fever, or hemoptysis.

Vital signs were notable for a blood pressure of 118/72 mm Hg and a heart rate of 84 beats per minute, with an oxygen saturation of 92% on pulse oximetry and a body mass index (BMI) of 16 kg/m². Examination revealed a cachectic male with poor dentition, subtle end-expiratory wheezing, and a grossly positive digital rectal examination. Admission laboratory results are summarized in Table 1, notable for severe anemia (hemoglobin 5 g/dL), marked neutrophilic leukocytosis, and hypoalbuminemia.

**Table 1:** Admission laboratory values INR: International Normalized Ratio.

Laboratory Test	Patient Value	Reference Range
Hemoglobin	5 g/dL	13.5-17.5 g/dL
Mean corpuscular volume (MCV)	97 fL	80-100 fL
Blood urea nitrogen (BUN)	39 mg/dL	7-20 mg/dL
Creatinine	1.3 mg/dL	0.6-1.3 mg/dL
Albumin	2.3 g/dL	3.5-5.0 g/dL
White blood cell count	18,000 /µL	4,000-11,000 /µL
Neutrophils	90%	40%-70%
Ferritin	600 ng/mL	24-336 ng/mL
Platelet count	157 ×10³/µL	150-400 ×10³/µL
INR	1.1	0.8-1.2
HIV fourth generation antigen/antibody test	Negative	Non-reactive

**Figure 1:**
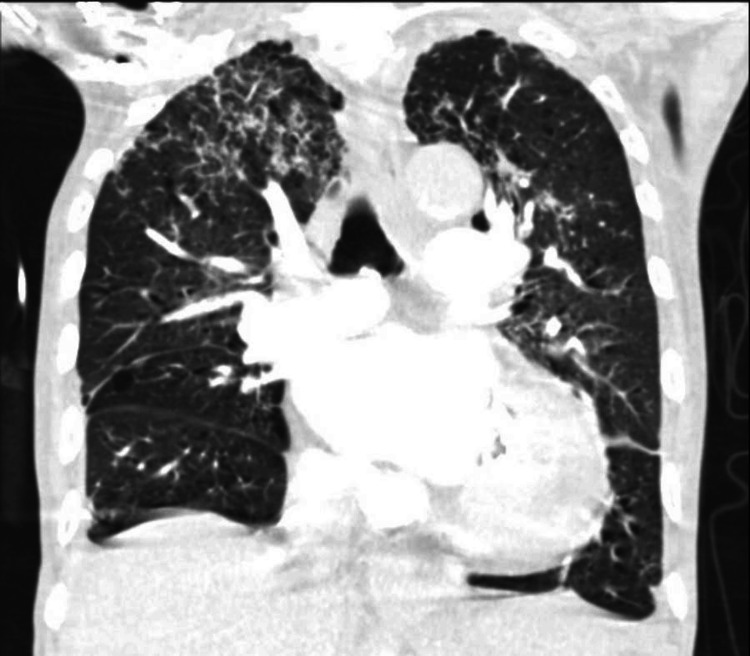
Coronal CT chest with upper-lobe predominant abnormalities

Additional diagnostics included a contrast-enhanced CT of the chest from his prior admission demonstrating multifocal bilateral pulmonary opacities, diffuse peribronchial thickening, small pleural effusions, and suspected distal airway mucus plugging. These findings were initially interpreted as most consistent with infectious or inflammatory bronchiolitis or aspiration pneumonitis (Figure 1). Subsequent microbiologic data available at the time of this admission included a sputum acid-fast bacilli (AFB) smear positive at 2+, with mycobacterial culture growing *Mycobacterium tuberculosis* complex after 10 days. Drug susceptibility testing demonstrated sensitivity to isoniazid, rifampin, ethambutol, and pyrazinamide.

**Figure 2:**
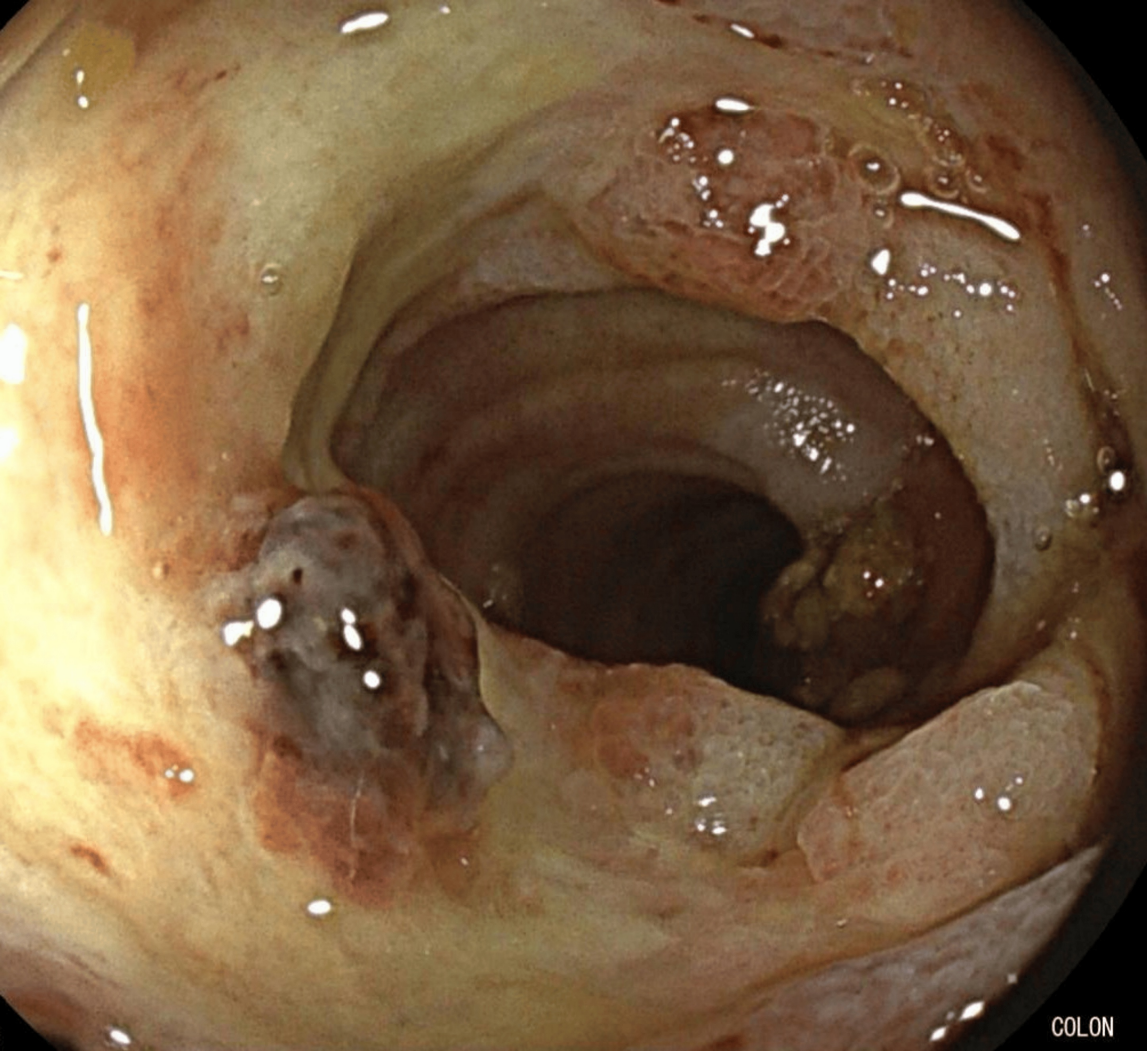
Terminal ileum ulcer with overlying visible vessel Endoscopic images obtained during colonoscopy demonstrating a large, semi-circumferential ulcer in the terminal ileum with an irregular base and an overlying visible vessel, with surrounding inflammation.

The patient was admitted for management of a presumed lower gastrointestinal bleed. He was hemodynamically stable despite presenting hematochezia and severe anemia. He was resuscitated with two units of packed red blood cells, with improvement in hemoglobin. Deliberate intubation of the terminal ileum revealed a large semi-circumferential ulcer measuring approximately 3 × 4 cm with a non-bleeding visible vessel (Figure 2). The visible vessel was treated with bipolar cautery with successful immediate hemostasis. No further bleeding was observed following endoscopic intervention. No other active colonic bleeding source was identified, aside from large internal hemorrhoids without active bleeding.

**Figure 3:**
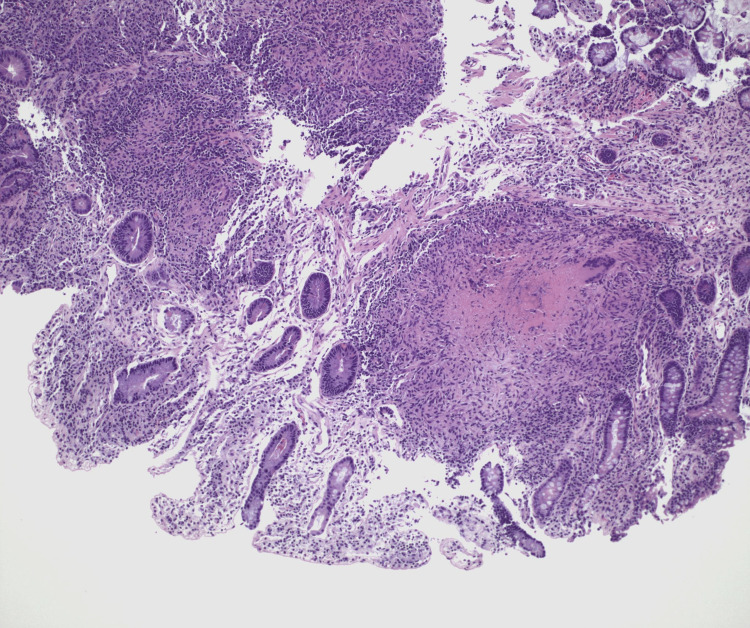
Granulomatous inflammation with central necrosis on hematoxylin and eosin staining

**Figure 4:**
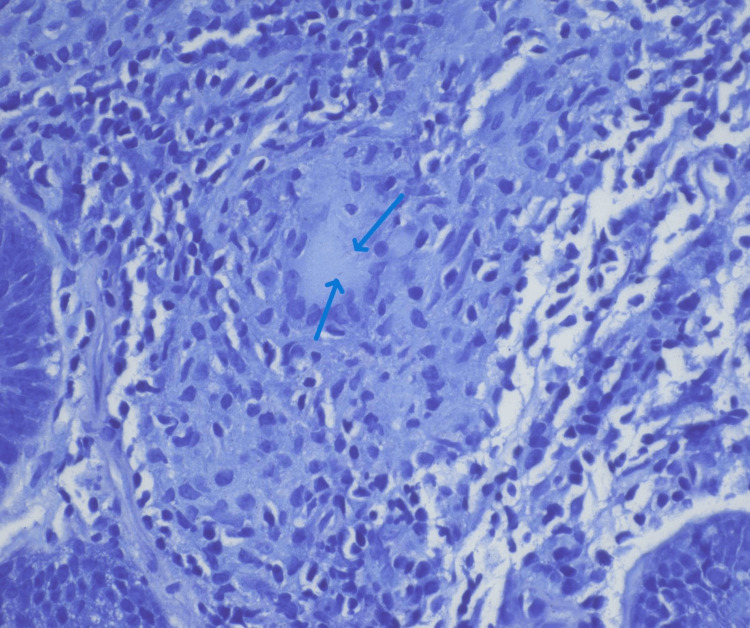
Acid-fast staining demonstrating organisms within multinucleated giant cells, consistent with tuberculosis

Targeted biopsies of the ulcer demonstrated chronic granulomatous inflammation with focal necrosis (Figure 3). The specimen was sent off-island for external pathology review, where acid-fast bacilli staining identified organisms consistent with mycobacterial infection, supporting the diagnosis of intestinal tuberculosis (Figure 4). Mycobacterial culture and nucleic acid amplification testing (NAAT) were not performed on the tissue specimen, which is acknowledged as a limitation given current American Thoracic Society (ATS)/Centers for Disease Control and Prevention (CDC)/Infectious Diseases Society of America (IDSA) recommendations.

## Discussion

Intestinal tuberculosis is an uncommon manifestation of extrapulmonary tuberculosis and is rarely included in the initial differential diagnosis of gastrointestinal bleeding. If left unrecognized, caseating granulomatous inflammation may progress to transmural ulceration and tissue destruction. As this process extends deeper into the bowel wall, it can erode into submucosal and subserosal vessels, including branches of the mesenteric arterial system, resulting in significant or potentially life-threatening hemorrhage.

While granulomatous inflammation of the intestine has a broad differential diagnosis encompassing both infectious and non-infectious etiologies (Table 2), Crohn’s disease remains the most clinically consequential misdiagnosis, as inappropriate immunosuppression can lead to dissemination of unrecognized tuberculosis. In this case, necrotizing granulomatous inflammation with positive acid-fast bacilli staining favored a diagnosis of intestinal tuberculosis over Crohn’s disease [10]. This was further supported by concordant pulmonary microbiologic data.

**Table 2:** Intestinal granulomas and distinguishing histologic and clinical features TB: tuberculosis, AFB: acid-fast bacilli.

Etiology	Category	Typical Histologic Features	Clinical/Diagnostic Clues
Crohn’s disease	Non-infectious	Small, well-formed non-caseating epithelioid granulomas; microgranulomas; crypt-centered inflammation	Longitudinal ulcers, skip lesions, cobblestoning, perianal disease, chronic relapsing course; absence of AFB staining
Intestinal tuberculosis	Infectious	Large confluent granulomas with caseous necrosis; Langhans giant cells; often submucosal; AFB may be present	Transverse or circumferential ulcers; TB exposure or endemic region; positive AFB staining or mycobacterial testing; in this case, necrotizing granulomas and positive AFB staining

Misdiagnosis carries significant clinical risk, as immunosuppressive therapy for presumed inflammatory bowel disease can lead to dissemination of unrecognized tuberculosis and adverse outcomes. Accordingly, the identification of granulomas on biopsy should prompt evaluation for mycobacterial infection. Current guidelines recommend screening for latent or active tuberculosis prior to initiating immunosuppressive agents, including corticosteroids, thiopurines, and tumor necrosis factor inhibitors, using a tuberculin skin test (TST) or interferon-gamma release assay (IGRA), alongside chest imaging and clinical risk assessment.

TST and IGRAs are commonly used adjuncts in the evaluation of tuberculosis infection, although important distinctions exist. IGRA is generally preferred in individuals with prior BCG vaccination, as it is not affected by vaccination and offers improved specificity, particularly relevant given routine BCG vaccination in the Philippines. In contrast, TST may yield false-positive results due to cross-reactivity. Both tests have reduced sensitivity in immunocompromised patients and in extrapulmonary disease and may be falsely negative due to anergy; additionally, neither reliably distinguishes latent from active infection. Consequently, clinical suspicion for tuberculosis must remain high when evaluating extrapulmonary presentations.

Notably, the absence of classic constitutional and pulmonary symptoms, such as weight loss, fever, and hemoptysis, despite severe anemia and disseminated disease is clinically striking; the patient’s advanced frailty, severe malnutrition (body mass index, 16 kg/m²; and hypoalbuminemia), and underlying chronic obstructive pulmonary disease likely impaired host immunity and blunted symptom expression, potentially delaying recognition of disease severity. The marked neutrophilia initially raised concern for bacterial superinfection but was ultimately attributed to systemic inflammation in the setting of physiologic stress and active tuberculosis, as no alternative source was identified [11,12].

## Conclusions

Although active tuberculosis is uncommon in low-incidence settings, clinicians may encounter latent or active tuberculosis when caring for patients from endemic regions. This case highlights the diagnostic overlap between intestinal tuberculosis and conditions such as Crohn’s disease, the potential for atypical presentations in elderly patients lacking classic symptoms, and the limitations of screening tests, particularly in immunocompromised individuals, where interferon-gamma release assays are generally preferred over tuberculin skin testing in those with prior BCG vaccination. Awareness of these factors, along with appropriate diagnostic evaluation of granulomatous disease, is essential for internists, gastroenterologists, and infectious disease physicians managing patients with relevant epidemiologic risk factors.

## References

[1] Eraksoy H (2021). Gastrointestinal and abdominal tuberculosis. Gastroenterol Clin North Am.

[2] Chakaya J, Khan M, Ntoumi F (2021). Global Tuberculosis Report 2020: reflections on the global TB burden, treatment and prevention efforts. Int J Infect Dis.

[3] Gan H, Mely M, Zhao J, Zhu L (2016). An analysis of the clinical, endoscopic, and pathologic features of intestinal tuberculosis. J Clin Gastroenterol.

[4] Zeng J, Zhou G, Pan F (2023). Clinical analysis of intestinal tuberculosis: a retrospective study. J Clin Med.

[5] Cheng W, Zhang S, Li Y, Wang J, Li J (2019). Intestinal tuberculosis: clinico-pathological profile and the importance of a high degree of suspicion. Trop Med Int Health.

[6] Beppu K, Osada T, Matsumoto K (2009). Gastrointestinal tuberculosis as a cause of massive bleeding. Med Sci Monit.

[7] Shi XC, Zhang LF, Zhang YQ, Liu XQ, Fei GJ (2016). Clinical and laboratory diagnosis of intestinal tuberculosis. Chin Med J.

[8] Lu S, Fu J, Guo Y, Huang J (2020). Clinical diagnosis and endoscopic analysis of 10 cases of intestinal tuberculosis. Medicine (Baltimore).

[9] Ben Ismail I, Rebii S, Mouna M, Sghaier M, Yaich K, Zoghlami A (2024). Intestinal tuberculosis complicated with perforation in an immunocompetent patient: case report and review of the literature. Heliyon.

[10] Giouleme O, Paschos P, Katsaros M, Papalexi F, Karabatsou S, Masmanidou M, Koliouskas D (2011). Intestinal tuberculosis: a diagnostic challenge: case report and review of the literature. Eur J Gastroenterol Hepatol.

[11] Caraux-Paz P, Diamantis S, de Wazières B, Gallien S (2021). Tuberculosis in the elderly. J Clin Med.

[12] Brown I, Kumarasinghe MP (2018). Granulomas in the gastrointestinal tract: deciphering the Pandora's box. Virchows Arch.

